# PRECICE^®^ magnetically-driven, telescopic, intramedullary lengthening nail: pre-clinical testing and first 30 patients

**DOI:** 10.1051/sicotj/2016048

**Published:** 2017-03-06

**Authors:** Pablo Wagner, Rolf D. Burghardt, Stuart A. Green, Stacy C. Specht, Shawn C. Standard, John E. Herzenberg

**Affiliations:** 1 Clinica Alemana de Santiago, Universidad del desarrollo Av. Vitacura 5951 Vitacura, Santiago Chile; 2 Department of Orthopaedics, University Medical Center Hamburg-Eppendorf Martinistr 52 20246 Hamburg Germany; 3 Pediatric Orthopaedic Department, Schön Klinik Harlaching Harlachinger Str 51 81457 Munich Germany; 4 Department of Orthopedic Surgery, University of California Irvine CA USA; 5 International Center for Limb Lengthening, Rubin Institute for Advanced Orthopedics, Sinai Hospital of Baltimore 2401 West Belvedere Ave Baltimore MD 21215 USA

**Keywords:** Limb lengthening, Intramedullary nail, Bone lengthening, Limb length discrepancy, Magnet

## Abstract

*Introduction*: Femoral/tibial lengthening with a telescopic, magnetically-powered, intramedullary nail is an alternative to lengthening with external fixation.

*Methods*: Pre-clinical testing was conducted of the PRECICE in a human cadaver. A retrospective review of the first 30 consecutive patients who underwent unilateral lengthening was also conducted. Nail accuracy was obtained by comparing the amount of nail distraction to the final bone length achieved at the end of the distraction process. Relative standard deviation of accuracy was used to calculate nail precision.

*Results*: Devices performed successfully in a human cadaver. Thirty consecutive patients (10 females, 20 males; mean age, 23 years) with limb length discrepancy (LLD) were followed an average of 19 months (range, 12–24 months). Etiology included congenital shortening (14), posttraumatic deformities (7), Ollier disease (3), osteosarcoma resection (1), prior clubfoot (2), hip dysplasia (1), post-septic growth arrest of knee (1), and LLD after hip arthroplasty (1). Twenty-four femoral and eight tibial nails were implanted. Mean preoperative lengthening goal was 4.4 cm (range, 2–6.5 cm); mean postoperative length achieved was 4.3 cm (range, 1.5–6.5 cm). Average consolidation index was 36.4 days/cm (range, 12.8–113 days/cm). Mean nail accuracy was 97.3% with a precision of 92.4%. Average preoperative and 12-month postoperative Enneking scores were 21.5 and 25.3 (*p* < 0.001), respectively. The preoperative and 12-month postoperative SF-12 physical and mental component scores were not statistically different. Nine complications (nine limb segments) resolved: two partial femoral unions, two suspected deep vein thrombosis (DVT), one delayed tibial union, one fibular nonunion, one peroneal nerve irritation, one knee joint subluxation, and one confirmed DVT. Twenty-nine (91%) of 32 limb segments achieved successful bone healing without revision surgery.

*Discussion*: Limb lengthening with PRECICE is reliable, but larger trials with longer follow-up will reveal limitations. Implantable nails prevent problems associated with external fixation, such as muscle tethering and pin-site infections.

## Introduction

The classic limb lengthening technique has been the Ilizarov method using external fixation [1]. However, external fixation is associated with lengthy treatment duration, pin-site infections, and muscle tethering leading to decreased joint motion [2, 3]. Modern innovations such as lengthening over nail (LON), lengthening and then nailing, or lengthening and then plating have been developed to decrease external fixation time [4–6].

The next generation in this drive to improve limb lengthening was self-lengthening intramedullary nails. Examples are the Intramedullary Skeletal Kinetic Distractor (ISKD) (Orthofix, McKinney, TX, USA) [7, 8], Guichet nail (Medinov-AMP, Roanne*,* France) [9, 10], and Fitbone nail (Wittenstein intense GmbH, Ingersheim, Germany) [11]. The motivation for this evolution is to eliminate pin-site infections, soft-tissue contractures, pain, and scarring as well as to allow for greater acceptance by patients and faster rehabilitation [7, 9, 11, 12].

The ISKD and the Guichet nails are both unidirectional, mechanically-driven, ratcheting devices that lengthen through twisting motions (intentional or spontaneous) of the limb. Until 2011, the ISKD was the only intramedullary device that received the Food and Drug Administration (FDA) approval. It is no longer distributed in the US but is still available in other countries. The ISKD rotational actuated mechanisms were sometimes unreliable, resulting in many complications [12–15]. In contrast to the ISKD and Guichet nails [9, 16], Fitbone is powered by high-frequency, electric energy induced by an external transmitter through the skin above a subcutaneous receiver [11]. It is not FDA approved.

The PRECICE (NuVasive, Inc, San Diego, California, USA) is a magnetically-driven, titanium intramedullary nail [17–20]. It is designed for antegrade femoral insertion (piriformis or trochanteric entry), retrograde femoral insertion (straight or with a Herzog bend), and antegrade tibial insertion. It is bidirectional and is activated by applying a magnetic field generator (external remote controller [ERC]) to the skin, making the rate/rhythm of distraction controllable [17]. The ERC consists of two external magnets that rotate to create a magnetic field that causes the magnet inside the PRECICE nail to rotate in the same direction.The ERC has been shown to be accurate and precise [19].

Our objective is to report a cadaveric study and the results in the first 30 consecutive patients who underwent treatment at our center using the PRECICE.

## Materials and methods

We used the original version of the PRECICE, called P1, which was available in 10.7-mm and 12.5-mm diameters and had a maximum lengthening of 65 mm. It was modular, with the lengthening mechanism joined with a set screw to an extension rod (shortest length of 230 mm). A new PRECICE model (called P2) was released in January 2014 that is not modular (solid construction).

### Human cadaveric study

Two PRECICE nails (10.7-mm diameter, 23 cm length) were implanted into the tibia and femur of a female cadaveric specimen. At the time of the cadaveric study, the PRECICE nail had not yet been implanted in any human patient at any center. The ERC was used to achieve 10 mm of distraction under fluoroscopic control. After distraction, dissection was performed to confirm the bone gap. Our goals were to detect any mechanical problem with the nail, to determine whether the instrumentation worked in a clinical scenario, and to confirm that the nail was capable of distracting despite the resistance of soft tissues.

### Clinical study

After the preliminary cadaveric study and Institutional Review Board’s approval (Approval Number 1879), a retrospective review was conducted of the first 30 patients who underwent insertion of the PRECICE. Inclusion criteria were limb length discrepancy (LLD) greater than 1.5 cm, unilateral femoral and/or tibial lengthening, sufficient bone stock without active infection, and ability to comply with treatment requirements and follow-up visits. Exclusion criteria were inadequate bone diameter to allow insertion of at least a 10.7-mm implant, LLD less than 1.5 cm, and inappropriate lengthening conditions (e.g., unresolved poly-trauma, nonunited fracture, open wounds, ulcers, impassable intramedullary canal, significant angular deformity, body mass index >30 kg/m^2^, poor lower limb bone quality, conditions affecting bone metabolism, Paget’s disease, uncontrolled diabetes, thyroid disease, peripheral vascular disease, malignancy, multiple illnesses, systemic/local infections, pacemaker [using the ERC may interfere with the pacemaker]).

Patients were followed for a one-year minimum. We collected details regarding their age, gender, etiology, goal length, achieved length, nail accuracy (comparison of the amount of nail distraction to the final bone length achieved at the end of the distraction process), and precision (100 – standard deviation of accuracy). Maturation index was the number of days to achieve consolidation after completing the distraction phase divided by length achieved in cm (days/cm). Distraction index was length achieved divided by number of days of distraction (mm/days). Consolidation index was total duration required to achieve bone healing starting after surgery divided by achieved length in cm (days/cm). Indices were calculated for the entire group, femoral group, and tibial group. Complications were classified as bone or soft-tissue complications. Bone healing was defined as consolidation of three of four cortices. Nonunion was defined as absent bone healing in all four cortices at six months after the distraction phase. Partial union was defined as solid healing of only one or two of the four cortices six months after distraction was completed. Delayed healing was defined as absence of bone healing three months after distraction was completed and consolidation index greater than 100 days/cm. Quality of life questionnaires (SF-12 and Enneking scores [21]) were collected preoperatively and at 12 months postoperatively.

### Operative technique, postoperative course, and follow-up

The surgical procedure to implant the PRECICE has been described [17, 19]. Nails were distracted 1 to 2 mm in the operating room to ensure proper function. For posttraumatic cases, acute correction of angular deformities was performed prior to inserting the PRECICE nail. Postoperative distraction began five days after insertion for femora and seven days for tibiae. Femoral lengthening rate was 1 mm/day, and tibial rate was 0.75 mm/day. After the distraction phase, patients were instructed to bear up to 40 lbs of weight until sufficient regenerate bone was noted. Full weightbearing was allowed without assistive devices when three of four cortices were fully healed.

Follow-up visits occurred every two weeks during the distraction phase and monthly during the consolidation phase. Anteroposterior (AP) and lateral view radiographs of the bone segment were obtained at every visit to determine the distraction gap length and bone regenerate quality. After complete bone healing, follow-up visits occurred every six months.

### Statistical Analysis

All data were analyzed using SPSS Statistics for Windows version 17.0 (SPSS Inc., Chicago, IL). A *p*-value of less than 0.05 was considered statistically significant.

## Results

### Human cadaveric study

Successful 10-mm tibial and femora lengthenings were achieved. The 10-mm bone gap was confirmed by image intensifier and with direct dissection. Surgical implantation hardware functioned as designed. No mechanical or hardware complications occurred during lengthening.

### Clinical study

**Figure 1.**
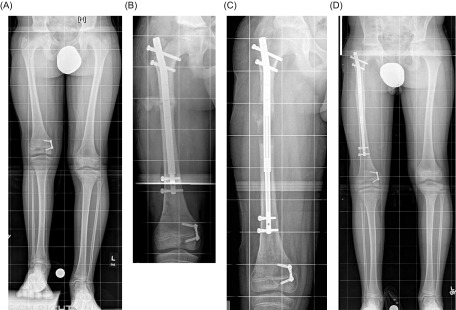
Clinical case shows normal healing. (A) Preoperative anteroposterior (AP) view full length standing radiograph of a patient with a limb length discrepancy. (B) AP view radiograph of the right femur after insertion of PRECICE. (C) AP view radiograph of the right femur after distraction. (D) Final AP view full length standing radiograph shows that the right femur healed after the lengthening goal was achieved. Figure used with permission (© 2016, Rubin Institute for Advanced Orthopedics, Sinai Hospital of Baltimore).

Thirty patients (24 femora, eight tibiae, 32 nails) were included. Two patients each had two nails inserted (ipsilateral simultaneous femoral plus tibial lengthenings). Ten female and 20 male patients (mean age, 23 years; range, 7–63 years) were followed for a mean of 19 months (range, 12–24 months). Etiology included fibular hemimelia/congenital femoral deficiency (14), seven posttraumatic deformities (7), Ollier disease (3), prior clubfoot (2), hip dysplasia (1), post-septic growth arrest (knee) (1), LLD after hip arthroplasty (1), and osteosarcoma resection (1). Mean goal length was 4.4 cm (range, 2–6.5 cm) and mean bone length achieved was 4.3 cm (range, 1.5–6.5 cm). Mean nail accuracy was 97.3% with a precision of 92.4% (Table 1) (Figure 1).

**Table 1.** Demographic information, nail accuracy/precision, and SF-12 scores.

Mean age (range)	23 years (7–63 years)
Gender	10 females
	20 males
Mean follow-up (range)	19 months (12–24 years)
Mean goal bone length (range)	4.4 cm (2–6.5 cm)
Mean achieved bone length (range)	4.3 cm (1.5–6.5 cm)
Mean nail accuracy	97.3%
Mean nail precision	92.4%
Mean Enneking scores:	
Preoperative score	21.5
12-month postoperative score (*p*-value)	25.3 (*p* < 0.001)
Mean SF-12 physical scores:	
Preoperative score	50.5
12-month postoperative score (*p*-value)	55.2 (*p* = 0.12)
Mean SF-12 mental scores:	
Preoperative score	48.4
12-month postoperative score (*p*-value)	57.5 (*p* = 0.2)

Mean consolidation index was 36.4 days/cm (range, 12.8–113 days/cm), mean maturation index was 22.4 days/cm (range, 4.2–96 days/cm), and mean distraction index was 0.64 mm/day (range, 0.35–1.25 mm/day) (Table 2). When differentiating between femora and tibiae, the distraction index was significantly different indicating our preference to distract faster in the femur (0.67 vs. 0.52, *p* = 0.002), but the consolidation and maturation indices did not achieve statistical significance (*p* = 0.09 and *p* = 0.12, respectively). This may be related to the small tibial group sample size.

**Table 2.** Consolidation, maturation, and distraction indices.

Index	All limb segments	Femora	Tibiae	*p* value
Consolidation index days/cm (range)	36.4 (12.8–113)	32.4 (12.8–113)	48 (22.4–101.3)	*p* = 0.09
Maturation index days/cm (range)	22.4 (4.2–96)	19.1 (4.2–96)	32 (5.9–80)	*p* = 0.12
Distraction index mm/day (range)	0.64 (0.35–1.25)	0.67 (0.4–1.25)	0.52 (0.36–0.69)	*p* = 0.002

Average preoperative and 12-month postoperative Enneking scores were 21.5 and 25.3 (*p* < 0.001), respectively. The SF-12 physical and mental component scores were not statistically different (Table 1) when comparing preoperative scores with the 12-month postoperative scores. This indicates no deterioration in patients’ perception of their condition. We advised elective nail removal for all patients after circumferential healing. In this series, 28 of 32 nails have been explanted.

**Figure 2.**
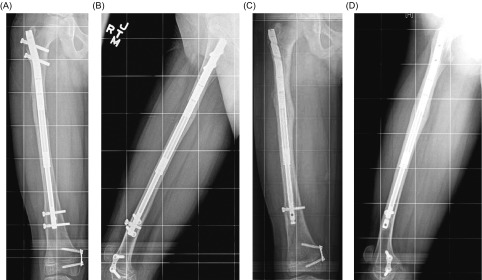
Clinical case with partial union. AP (A) and lateral (B) view radiographs of the right femur show partial union. After injection of bone marrow aspirate concentrate (platelet-rich plasma) into the partial union site and nail dynamization by removing the proximal locking screws, AP (C) and lateral (D) view radiographs showed complete healing. Figure used with permission (© 2016, Rubin Institute for Advanced Orthopedics, Sinai Hospital of Baltimore).

We observed nine complications in nine limb segments (28%). No nail-specific complications or infections occurred. Four bone healing complications occurred: one fibular nonunion (healed after bone graft application and internal fixation); two femoral partial unions (treatment explained in the following paragraph) (Figure 2); and one delayed tibial union healed without surgical intervention (consolidation index 113 days/cm with bone healing starting at four months). Five soft-tissue complications occurred: one knee posterolateral rotatory subluxation (required contracture release and extra-articular posterior cruciate ligament reconstruction); one peroneal nerve neuropathy (required peroneal nerve decompression); one deep vein thrombosis (DVT); and two leg swelling episodes that were admitted for pain control (one after rod insertion and one after rod removal; both were negative for DVT). None of the complications affected the lengthening goal.

In both cases of femoral partial unions, bone marrow aspirate was obtained and then concentrated using the BioCUE centrifuge system (Biomet, Warsaw, IN). The resulting bone marrow aspirate concentrate (BMAC) that contained platelet-rich plasma was injected into the area of delayed healing [22]. In one of the two cases of femoral partial union, the PRECICE nail was dynamized by removing the proximal locking screws to induce healing.

Two patients (two limb segments) achieved full bone healing as defined by this study (three of four cortices healed) and were fully weightbearing and asymptomatic. However, radiographs revealed a persistent isolated unhealed cortex. In both cases, fracture risk after PRECICE removal was discussed, patients underwent autologous bone grafting, and successful and complete bone healing was achieved. Twenty-nine (91%) of 32 limb segments achieved successful bone healing without a revision surgery.

## Discussion

Limb lengthening has evolved from using external fixation to intramedullary devices. External fixation is still the treatment of choice in some cases, such as children with open growth plates in the tibia. Our series included 12 skeletally immature children with open growth plates. In all 12, the PRECICE was inserted across the greater trochanteric apophysis via a careful trochanteric femoral approach to avoid the femoral neck base. We consider it a contraindication to insert PRECICE across open distal femoral or proximal tibial epiphyses.

Many reports describe good clinical results of the Guichet nail [9, 10], Fitbone [11, 23–26], and ISKD [7, 15]. However, most of the reports describe important limitations and complications. The complication rate in our series was 28%, and none resulted in long-term sequelae. This compares favorably with reported complication rates of 22–80% in other intramedullary lengthening devices [7, 9–11, 15, 23–26].

Most limbs achieved the desired goal length in the ISKD studies, but complications were frequent (50–80%) [10, 13, 14, 26], sometimes more frequent than with LON [27]. These complications were mainly associated with the inability to control the lengthening rate, which can result in additional surgery.

The Guichet nail complication rate ranges from 22 to 39% [9, 16]. The required leg rotation to distract has led to increased pain and occasional need for hospital re-admission to perform leg rotations under anesthesia [9, 16]. The Fitbone has shown good results and good control over the lengthening speed. However, a high complication rate (31–62%) has been reported [23–27] (e.g., broken/infected subcutaneous actuator wires, motor dysfunction requiring secondary surgery). These complications were not observed in our series. Additionally, the Fitbone, ISKD, and Guichet devices are not available for routine use in the US, are not available in the US, or are not FDA cleared.

The PRECICE nail was designed to address complications that occur with other lengthening nail systems, such as runaway nails or premature consolidation (e.g., ISKD, Guichet) and electrical wire fractures (e.g., Fitbone). The PRECICE showed comparable results with other intramedullary nails reported in the literature. Our population was relatively young, and 91% of bone segments achieved successful bone healing without revision surgery. No hardware breakage or failures were observed in our study they but have been reported with other nails in up to 28% of cases [9, 10, 13, 14, 16, 23–27]. Other studies have reported mechanical failures and breakage with the first generation of the PRECICE (the P1 system). Schiedel et al. [18] conducted a study of 26 limb segments. Two nails broke during the consolidation phase (one had fatigue failure along the welding seam and the other broke between the lengthening unit and the extension rod when the patient fell). In addition, two nails did not function. The nail was exchanged in one case, and in the other case, the surgical procedure was changed and the patient was excluded from the study. Tiefenböck and Wozasek [28] observed one tibial case in which the PRECICE broke at the welding seam 15 months after implantation and created a 15° valgus and procurvatum deformity. Kirane et al. [19] reported that one of the 25 PRECICE nails had a nonfunctional distraction mechanism. The nail was exchanged and the segment achieved the lengthening goal. Breakage of IM nails is more likely with weightbearing prior to consolidation. This scenario is most often seen with bilateral lengthenings, as in lengthening for dwarfism or stature. The welding seam seems to have been a weak point of the P1 system, but this has been modified in the second generation of the PRECICE (P2 system) to make it stronger.

The average lengthening achieved using the ERC is comparable to those of other studies (43 mm) [7, 9, 16, 23, 24, 27] with an excellent nail accuracy and precision. In our series, the precision of the PRECICE is confirmed, being at least as good as the Fitbone, Guichet, and ISKD, given that no cases of runaway nails or premature consolidation were observed (no specific precision and accuracy data for Fitbone, Guichet, and ISKD nails were found in the literature). One advantage the PRECICE nail has over the ISKD is the ability to change the amount of distraction desired postoperatively. With the ISKD, the physician must decide the final distraction amount in the operating room. Slowing or stopping ISKD lengthening requires bracing, casting, external fixation application, and/or ISKD removal. The daily length obtained varied greatly. The PRECICE is the only intramedullary lengthening nail that is reversible.

Another advantage of the PRECICE is that the lengthening rate and rhythm are easily controlled, similar to external fixation. The lengthening speed can be increased to stave off premature consolidation or decreased to prevent nonunion. The consolidation index for the PRECICE nail is similar to that reported in the Fitbone and Guichet literature [9, 16, 23, 24, 26]. However, the PRECICE consolidation index (36 days/cm) is better when compared to the ISKD (44 and 50 days/cm) [10, 26]. The maturation and distraction indices were comparable between all nails.

Functional scores obtained preoperatively and postoperatively show a slight improvement in the quality of life on the Enneking score, but it is not evident with the SF-12 score. Functional scores are not commonly reported in the current literature for motorized nails. Hankemeier published Enneking scores for the ISKD in four patients, having a 14-month postoperative score of 26.8 points [12], which is similar to our results. For the Fitbone, Dinçyürek et al. [26] used a non-validated functional score that showed excellent results in all 14 patients, while García-Cimbrelo et al. [16] reported 22 of 24 patients with excellent scores using the same non-validated functional score for the Guichet nail.

No hardware complications or infections were noted in our series. Nail failures (e.g., failure to lengthen, nail fracture, wire fracture/infections surrounding the Fitbone antenna) are common with some available nails. Bone healing impairment was noted in four (13%) bone segments including one symptomatic fibular nonunion, one tibial delayed union, and two femoral partial unions. We created this new concept of “partial union”, as these limbs differ from circumferential nonunions because they have a bone bridge (albeit incomplete) and do not have delayed union (they have bone healing at an appropriate speed). Partial unions have appropriate bone healing, but not in the complete bone circumference (Figure 2). Our protocol calls for unrestricted weightbearing only after consolidation of at least three cortices. We have limited experience in this series with partial union or nonunion; however, in cases of persistent partial union (1–2 cortices only), we recommend autologous bone grafting, bone marrow aspirate concentrate (platelet-rich plasma), and/or nail exchange (trauma nail). For persistent, long-term nonunion of a single cortex we recommend bone grafting, bone marrow aspirate concentrate (platelet-rich plasma), nail dynamization, and/or nail exchange. We expect that this approach to partial union and nonunion will be further refined as our experience with this device grows and additional reports are published in the literature. We recommend eventual PRECICE removal for all patients, provided there is 360° healing of the regenerate.

The limitations of this study are that it is a retrospective case series without a comparison group and that it represents a heterogeneous group (e.g., age of the patients, femoral and tibial treatment). More follow-up is needed, as well as a larger sample size. The strengths of this study are that the average follow-up is 19 months, 28 of 32 nails have been explanted, and no patients were lost to follow-up.

## Conflict of interest

Please note that two authors share the same initials. We distinguished between them by stating their placement in the author list (fourth author versus fifth author in the list).

The institution of JEH and SCS (fifth author) received a research grant from NuVasive to conduct this study.

SAG, SCS (fifth author), and JEH have or may receive payments or benefits from NuVasive.

SCS (fifth author) has or may receive payments or benefits from Pega Medical.

JEH has or may receive payments or benefits from OrthoPediatrics, Orthofix, and Smith & Nephew.

PW, RDB, and SCS (fourth author) certify that they do not have any financial conflict of interest (e.g., consultancies, stock ownership, equity interest, patent/licensing arrangements) in connection with this article.

The institution of SCS (fifth author) and JEH has received funding from: Baxter, CS Medical Supply, DePuy Synthes, Merete Technologies, Metro Prosthetics, MHE Coalition, NuVasive, Orthofix, OrthoPediatrics, Smith & Nephew, Stryker, and Zimmer Biomet.
